# Renal Afferents

**DOI:** 10.1007/s11906-016-0676-z

**Published:** 2016-09-05

**Authors:** Alissa A. Frame, Casey Y. Carmichael, Richard D. Wainford

**Affiliations:** Department of Pharmacology & Experimental Therapeutics and The Whitaker Cardiovascular Institute Boston University School of Medicine, 72 East Concord St, Boston, 02118 MA USA

**Keywords:** Renal afferent nerves, Renal efferent nerves, Hypertension, Radiofrequency ablation, Renal denervation, Sympathetic nervous system

## Abstract

**Purpose of Review:**

The etiology of hypertension, a critical public health issue affecting one in three US adults, involves the integration of the actions of multiple organ systems, including the renal sympathetic nerves. The renal sympathetic nerves, which are comprised of both afferent (sensory input) and efferent (sympathetic outflow) arms, have emerged as a major potential therapeutic target to treat hypertension and disease states exhibiting excess renal sympathetic activity.

**Recent Findings:**

This review highlights recent advances in both clinical and basic science that have provided new insight into the distribution, function, and reinnervation of the renal sympathetic nerves, with a focus on the renal afferent nerves, in hypertension and hypertension-evoked disease states including salt-sensitive hypertension, obesity-induced hypertension, and chronic kidney disease.

**Summary:**

Increased understanding of the differential role of the renal afferent versus efferent nerves in the pathophysiology of hypertension has the potential to identify novel targets and refine therapeutic interventions designed to treat hypertension.

## Introduction

Hypertension, which affects one third of the US adult population and approximately 20 % of adults worldwide, is a major risk factor for adverse cardiovascular events—significantly contributing to the epidemic of cardiovascular and kidney disease, stroke, premature death, and disability [1]. Hypertension is a complex multifactorial disease involving the integration of multiple regulatory systems and our understanding of the pathogenesis of this disease state remains incomplete. Recent studies have explored the renal sympathetic nerve-mediated mechanisms, both afferent and efferent nerve based, in the long-term regulation of blood pressure as increasing basic science and clinical evidence implicates the renal nerves as a contributor to the pathophysiology of hypertension [2–4]. Removal or interruption of the afferent and efferent renal sympathetic nerves, which are located together in the adventitia of the renal arteries, has been shown to reduce blood pressure through multiple mechanisms. Reduction in efferent renal nerve traffic to the kidney prevents sympathetic nervous system-mediated (1) increased activity of the renin-angiotensin-aldosterone system, (2) renal sodium reabsorption, and (3) reduced renal blood flow [5]. In contrast, removal of afferent renal nerve traffic impacts the reno-renal reflexes to modulate central sympathetic outflow to peripheral organs (e.g., kidneys, heart) and the vasculature. The sensory renal afferent nerves, located predominantly in the renal pelvis [5], are chemo- and mechanosensitive and upon activation can elicit either (1) an inhibitory reno-renal reflex to evoke renal sympathoinhibition [5, 6] or (2) an excitatory reno-renal reflex to evoke sympathoexcitation [6–8]. Highlighting the potential importance of the renal sympathetic nerves in regulating blood pressure, it has been suggested that more than 50 % of clinical hypertension cases can be categorized as neurogenic essential hypertension and feature impaired regulation of the sympathetic nervous system [9]. Therefore, a more complete understanding of the role(s) of the afferent renal nerves, which have a profound impact on central sympathetic outflow, in both health and disease will potentially reveal novel targets and treatment paradigms for hypertension. The purpose of this review is to highlight recent advancements in our understanding of the actions of the afferent renal nerves in the etiology and pathogenesis of hypertension.

## Catheter-Based Radiofrequency Renal Denervation

The use of catheter-based radiofrequency renal denervation (RFRD), which interrupts both the afferent and efferent renal nerves, as an interventional approach to treat hypertension has been under investigation in multiple clinical trials for the past several years. The SYMPLICITY HTN-1 and HTN-2 trials reported the blood pressure-lowering effects of RFRD are sustained for at least a 36-month period in relatively small populations with severe treatment resistant hypertension [4, 10]. In contrast, the SYMPLICITY HTN-3 trial failed to show a significant reduction in systolic blood pressure between sham and RFRD groups 6 months post treatment [11], highlighting the pressing need to deepen our understanding of the roles of the afferent and efferent nerves in blood pressure regulation. The SYMPLICITY HTN-3 trial has several advantages over SYMPLICITY HTN-1 and HTN-2, including a larger sample size, inclusion of a control sham procedure, and a single-blinded experimental design. This raises the possibility, which remains to be fully investigated, that the long-term antihypertensive effects observed in the highly controlled small-scale HTN-1 and HTN-2 trials may not be reproducible in the general population. The issues relating to the design and conductance of SYMPLICITY HTN-3 are beyond the scope of this review—however, the effectiveness of the RFRD procedures conducted in this study have significant implications for the reported negative outcome. In this study, on average, operators performed approximately 3 RFRD procedures without previous experience. This raises the significant issue that lack of operator experience may have impacted the results through incomplete denervation [12], a profound issue given the lack of a standardized accepted test for the effective destruction of the renal sympathetic nerves during the procedure [13]. Furthermore, the DENERHTN trial, in which RFRD plus a standardized stepped-care antihypertensive treatment (SSAHT) decreased blood pressure more than identical SSAHT alone, highlights the potential importance and clinical applicability of RFRD as a therapeutic approach [14••]. Ultimately, the interpretation of conflicting data from clinical RFRD trials is complicated by the current absence of a clinical test confirming the effective destruction of the renal sympathetic nerves [13], and the degree of denervation required for therapeutic benefit in humans has not been investigated.

Subsequent post hoc analysis of the SYMPLICITY HTN-3 trial has revealed important predictors of the blood pressure response to RFRD which are likely to guide future investigations pursuing RFRD. Supporting the impact of patient medication, as revealed in DENERHTN, aldosterone antagonist use and non-use of vasodilators was a positive predictor of RFRD success. Highlighting the pivotal role of how RFRD is performed, these analyses revealed that a higher number of radiofrequency ablations and RFRD delivered in a four-quadrant pattern resulted in greater blood pressure reduction [15•]. Two recent basic science studies have suggested that modifications to the RFRD technique may improve efficacy of the procedure. An elegant study performed in swine conducted RFRD at three sites, near the ostium, in the main renal artery near the bifurcation, and in the renal artery extrarenal branches [16]. This study revealed the greatest reduction in renal NE content was observed following RFRD in the extrarenal arterial branches, a result that may reflect that this location had the greatest density of renal nerves and the greatest number of nerves in proximity to the lumen. A second study conducted in dogs suggests that electrical stimulation of the renal nerves, which evoked rapid changes in blood pressure via central mechanisms, could represent a response that could (1) identify the sites at which RFRD should be applied and (2) be applied post RFRD to confirm efficacy of RFRD [17••]. The validation of such a test is critical given the conflicting results of clinical RFRD trials and the observation that a threshold of 90 % surgical renal denervation is required for therapeutic benefit in animal models. At present it remains unknown if more extensive RFRD of both the afferent and efferent renal nerves and conductance of RFRD closer to the kidney would result in more profound and consistent reductions in blood pressure. However, increased ablation sites or increased energy frequency may evoke adverse effects not observed under the current treatment protocol.

## Renal Afferent and Efferent Nerve Distribution and Reinnervation

The unmet need to better understand the physiology and anatomy of the renal sympathetic nerves and characterize the impact of reinnervation post renal sympathetic nerve denervation has led to several recent advances. A pioneering study conducted in human autopsy subjects has shed new insight into the anatomical distribution of the peri-arterial sympathetic renal nerves located around the renal arteries [18]. This important study has revealed the anatomical distribution of the renal nerves with a clear predominance of efferent versus afferent nerve fibers and a decreasing presence of nerve fibers from proximal to distal locations. Further, it was revealed that within the distal regions (closest to the kidney), approximately 75 % of nerves are within 3 mm of the lumen, well within the range of RFRD, which generates lesions of 4-mm diameter. This suggests, as confirmed in a recent study conducted in swine [16], that greater RFRD efficacy is likely when ablation is conducted distally. Significantly, there was a difference observed in renal nerve anatomy between normotensive versus hypertensive subjects [18]. Two recent postmortem studies support the hypothesis that in renal arteries with a large diameter, renal nerve innervation is located further from the lumen, which would reduce efficacy of RFRD and require greater energy to produce significant ablation [19, 20]. The most recent study [20] again supports the growing body of evidence that the nerves are closer to the lumen in distal versus proximal regions. This increased knowledge of renal nerve anatomy will facilitate the refinement of current RFRD techniques, with future directions including RFRD at more distal locations and the adoption of a circumferential versus interrupted pattern of ablation.

As it is not yet possible to assess reinnervation in human subjects, a recent study conducted in normal Sprague-Dawley rats utilized immunohistochemistry to investigate the time course of renal reinnervation following surgical renal denervation (RDN) [21]. In this study, peptide markers of both afferent (substance P, calcitonin gene-related peptide) and efferent renal nerves (tyrosine hydroxylase, neuropeptide Y) were restored 9–12 weeks post denervation. While these results should be confirmed using neuroanatomic markers to verify the renal nerves as the source of the peptide markers, this study raises the intriguing, yet to be addressed, question of the functionality of the renal nerves following reinnervation in this setting. A recent study in sheep partially addresses this question by reporting that physiological responses to electrical stimulation of the renal nerves are abolished immediately following RFRD in normotensive sheep but are restored at 5.5 and 11 months post RFRD. Importantly, these functional changes are mirrored by the initial loss and subsequent restoration of peptide markers of both afferent and efferent renal nerves [22•]. Given the prolonged time frame of blood pressure reduction post RFRD in human patients [4, 10], it is highly likely that reinnervation has occurred without altering the blood pressure phenotype. A significant and clinically relevant question that remains unanswered is the functional status of reinnervated nerves. Are the responses of the regrown renal afferent nerves restored to normal in hypertensive animals or human subjects? Do regrown efferent nerves exhibit normal regulation of sodium, renin, and renal hemodynamics? Is there resetting of central nervous system mechanisms to alter sympathetic outflow following RFRD?

## Selective Renal Afferent Nerve Ablation

As previously discussed, surgical denervation and RFRD removes the influence of both the afferent and efferent renal sympathetic nerves—preventing the dissection of the individual actions of each arm. To facilitate the dissection of the role of the renal afferent nerves, a novel method of selective ablation of the renal afferent nerves by periaxonal application of capsaicin has been developed in rats [23••]. This technique selectively disrupts the renal afferent but not efferent renal nerves (confirmed pharmacologically and immunohistochemically), and supports recent studies on the timeline of nerve regrowth, with afferent nerve reinnervation suggested by the return of calcitonin gene-related peptide to 50 % of control levels within 7 weeks [23••]. However, the time course of renal afferent nerve reinnervation requires further investigation using neuroanatomical markers. These studies provide a new opportunity to examine the contribution of the afferent renal nerves to multiple pathophysiological states. Studies conducted using this technique suggest that the renal afferent nerves are not essential to maintain normotension and sodium balance in response to graded chronic alterations in dietary sodium intake Sprague-Dawley rats. In contrast, the renal afferent nerves play a role in the full development of deoxy-corticosterone acetate-salt hypertension, with afferent nerve ablation attenuating the observed hypertension by approximately 50 % [23••]. Extending investigations into the role(s) of the renal sympathetic nerves in the development of salt-sensitive hypertension, bilateral RDN conducted after the establishment of hypertension reduces blood pressure in the Dahl salt-sensitive (DSS) rat model [24]. A follow-up study confirmed that bilateral RDN reduces blood pressure in established short- and long-term DSS hypertension, and that this response reflects an efferent renal nerve effect as renal afferent nerve removal did not reduce blood pressure in hypertensive DSS rats [25]. These new studies, extending findings from the 1980s in which bilateral RDN prior to high salt intake did not attenuate the development of DSS hypertension, suggest that the underlying cause of hypertension, and the role of the renal nerves, may be different during developing versus established hypertension.

Bilateral RDN, but not selective renal afferent nerve ablation, attenuates angiotensin-II hypertension and inflammation, further suggesting that the differential impact of the afferent versus efferent nerves on blood pressure regulation may depend on the underlying cause of hypertension [26]. The differential responses observed to RDN and selective renal afferent nerve ablation in these recent studies highlight the unique mechanistic information that can be derived using the novel renal afferent ablation technique, and future studies are required to assess the roles of the renal afferent nerves in other hypertension paradigms (e.g., obesity-induced hypertension, neurogenic hypertension) and settings involving excess sympathetic nerve activity (e.g., heart failure).

## The Renal Afferent Nerves and Heart Failure

In the pathophysiology of heart failure, which can arise as a result of hypertension-evoked heart disease, reduced cardiac output and organ perfusion evokes multiple compensatory mechanisms including activation of the sympathetic nervous system. Increased efferent renal nerve activity, which evokes renal sodium and water retention, renin release, and renal vasoconstriction, is widely accepted to contribute to renal dysfunction in heart failure [27]. Further, recent studies in conscious sheep have identified the hypothalamic paraventricular nucleus (PVN) as a central site that regulates increased renal sympathetic outflow in heart failure [28]. In contrast, the role(s) of the renal afferent nerves in the pathophysiology of heart failure remains largely unexplored. Recent studies from the Patel laboratory reported that there is increased activity of RVLM-projecting PVN neurons in a rat model of chronic heart failure [29]. Advancing this work, the same authors have revealed that there is a neural connection from the PVN to the RVLM that is activated by direct stimulation of the renal afferent nerves [30•]. These data illustrate that afferent output from the kidney is integrated with PVN preautonomic neurons, significantly enhancing our mechanistic understanding of the actions of the renal afferent nerves. This raises the untested hypothesis that this novel renal afferent nerve activated neural circuit may play a critical role in the modulation of sympathetic outflow in heart failure. It has been previously established that the inhibitory mechanoreceptor reno-renal reflex is attenuated in heart failure [5]. However, the modulation of the excitatory chemo-reflex in heart failure remains to be established.

The impact of RDN on heart failure has been investigated in multiple experimental models and in clinical studies in the last several years. Post-myocardial infarction bilateral RDN improves cardiac function in rats [31]. In rat models of heart failure, bilateral RDN has been demonstrated to (1) attenuate renal sodium reabsorption via a Na-K-2Cl dependent pathway when RDN is performed prior to the establishment of heart failure [32] and (2) improve cardiac function when RDN is conducted during established heart failure [33]. In larger animal models of pacing-induced heart failure, bilateral RDN prior to heart failure induction improved cardiac function [34]. Suggesting the potential importance of bilateral RDN, unilateral RDN did not improve cardiac function despite improving cardiac autonomic balance via reductions in sympathetic outflow in a rabbit pacing-induced heart failure model [35]. In clinical trials in heart failure patients, RDN has been demonstrated to be safe and efficacious and to increase walking distance [36], reduce left ventricular mass, and increase ejection fraction [37, 38]. Collectively, these basic science and clinical data suggest that RDN represents a therapeutic approach that would benefit heart failure patients.

Ventricular arrhythmias (VAs) are the major cause of sudden cardiac death in most forms of heart disease, including hypertension-evoked heart disease [39]. It is well established that increased sympathetic activity contributes to the development and maintenance of VAs [40]. As such, recent studies have investigated the utility of RFRD, which reduces sympathetic activity, as a therapeutic approach to treat VAs. A pivotal role of the renal nerves in triggering VAs has been demonstrated by evidence that electrical activation of the renal sympathetic nerves promotes acute ischemia-induced VAs through actions on the left stellate ganglion in a canine model [41]. Further evidence generated in canine models supports a role of the renal nerves in VAs, as RDN reduces VAs during acute myocardial ischemia [42] and pacing-induced heart failure [34]. These studies suggest the mechanism underlying the attenuation of VAs following RDN is a correction of ventricular electrophysiological remodeling [34, 42]. The utility of RDN in the clinical setting as an approach to treat VAs has been explored in small populations with cardiac myopathy and treatment-resistant ventricular tachycardia. These studies have demonstrated (1) the safety of this procedure in this patient population and (2) a profound reduction in VAs [43–45]. Collectively, these data suggest that RDN represents a potential approach to treat heart failure and VAs, and that patients with identified sympathetic over activity are likely to benefit most from this approach. However, further studies to elucidate the likely differential effects of the afferent versus efferent renal nerves in these clinical paradigms would provide additional mechanistic insight.

## The Renal Afferent Nerves and Obesity-Induced Hypertension

It is well established that increased sympathetic nervous system activity contributes to the development and maintenance of obesity-induced hypertension [46]. Of relevance to this review is the pivotal role of the renal sympathetic nerves in obesity-evoked hypertension. Bilateral RDN restored blood pressure to normotensive levels in dogs with established obesity-induced hypertension [47]. Significantly, confirming the validity of a catheter-based approach, RFRD lowered blood pressure in a dog model of obesity-induced hypertension despite only partial nerve ablation [48]. Based on seminal work done in the 1990s in the laboratory of Dr. Hall, it is thought that the major benefits of RDN in obesity-induced hypertension are mediated via efferent renal nerve removal versus interruption of afferent renal nerve pathways. Recent findings in obese normotensive Sprague-Dawley rats imply that neural signals originating from the kidney (presumably renal afferent nerve mediated) contribute to autonomic dysregulation (i.e., impaired arterial and cardiopulmonary baroreflexes) in obesity [49]. While a direct role of the afferent renal nerves in mediating signal transduction from the kidney in this paradigm requires further confirmation (e.g., selective renal afferent nerve ablation), these studies challenge the lack of a role of the renal afferent nerves in this pathophysiological condition and suggest reassessment of the impact of the renal afferent nerves in obesity-induced hypertension may yield new insight.

## The Renal Afferent Nerves and Chronic Kidney Disease

Chronic kidney disease (CKD), which affects approximately 8 % of the world’s population over the age of 30, can be initiated or exacerbated by hypertension. CKD exhibits the hallmarks of sympathetic activation and baroreflex dysfunction that increases with disease progression [50]. Recent studies in a rodent model of cisplatin-induced renal failure have reported inappropriate activation of renal afferent sensory nerves, which impairs the regulation of renal sympathetic outflow. The authors report that high and low pressure baroreflex regulation of renal sympathetic nerve activity and volume expansion evoked renal sympathoinhibition is attenuated in cisplatin-induced renal failure—responses that are restored following acute bilateral RDN [6, 51]. These data suggest that in CKD, renal afferent sensory nerve activation via inflammatory pathways may evoke an excitatory reno-renal reflex to enhance renal efferent nerve activity and renal sympathoexcitation. Extending these data to a clinical setting, patients with end-stage renal disease on dialysis exhibit a marked increase in renal nerve density versus control subjects. Additionally, this study reported increased renal nerve endings present in patients with hypertensive arteriolar damage [52•]. This study did not differentiate between the presence of renal afferent versus efferent fibers but suggests RFRD may be a suitable therapeutic approach in the setting of CKD.

## Conclusion

Over the last several years, our understanding of the locations and function of the renal afferent and efferent nerves has dramatically increased through novel discoveries in both basic science and clinical arenas. These findings have solidified the renal sympathetic nerves as a predominant mechanism in multiple hypertensive disease states to drive elevations in both sympathetic outflow and blood pressure. The advances outlined in this review have revealed an increasingly important role of the renal sympathetic nerves in blood pressure regulation and sympathetic outflow. These findings suggest that the site at which RFRD is performed may significantly impact the outcome in terms of blood pressure, and that the renal afferent nerves may function differently depending on the underlying causes of hypertension. The next few years will provide further delineation of the role(s) of renal afferent nerve pathways and will potentially identify cross talk of these pathways across hypertension disease states. We speculate that current and future identification of the function and anatomy of the renal afferent and efferent sympathetic nerves will generate new therapeutic targets and refinements to current catheter-based renal denervation approaches that will have broad applications in the treatment of human hypertension.
